# A blinded evaluation of the efficacy and safety of glycopyrronium, a once-daily long-acting muscarinic antagonist, versus tiotropium, in patients with COPD: the GLOW5 study

**DOI:** 10.1186/1471-2466-14-4

**Published:** 2014-01-17

**Authors:** Kenneth R Chapman, Kai-Michael Beeh, Jutta Beier, Eric D Bateman, Anthony D’Urzo, Robert Nutbrown, Michelle Henley, Hungta Chen, Tim Overend, Peter D’Andrea

**Affiliations:** 1 Asthma and Airway Centre, University Health Network, Toronto Western Hospital, Rm 7-451 East Wing, 399 Bathurst Street, Toronto, ON, Canada; 2 Insaf Respiratory Research Institute, Wiesbaden, Germany; 3 University of Cape Town, Cape Town, South Africa; 4 Department of Family and Community Medicine, University of Toronto, Toronto ON, Canada; 5 Novartis Horsham Research Centre, West Sussex, UK; 6 Novartis Pharmaceuticals Corporation, East Hanover, NJ, USA

**Keywords:** COPD, Glycopyrronium, Breezhaler, Tiotropium, Bronchodilator, Long-acting muscarinic antagonist, Blinding

## Abstract

**Background:**

Two once-daily long-acting muscarinic antagonists (LAMAs) are currently available for the treatment of chronic obstructive pulmonary disease (COPD) – tiotropium and glycopyrronium. Previous studies have compared glycopyrronium with open-label tiotropium. In the GLOW5 study, we compare glycopyrronium with blinded tiotropium.

**Methods:**

In this blinded, double-dummy, parallel group, 12-week study, patients with moderate-to-severe COPD were randomized 1:1 to glycopyrronium 50 μg once daily or tiotropium 18 μg once daily. The primary objective was to demonstrate the non-inferiority of glycopyrronium versus blinded tiotropium with respect to trough forced expiratory volume in 1 second (FEV_1_) following 12 weeks of treatment (non-inferiority margin: –50 mL). Secondary objectives were to evaluate glycopyrronium versus tiotropium for other spirometric outcomes, breathlessness (Transition Dyspnea Index; TDI), health status (St George’s Respiratory Questionnaire; SGRQ), daily rescue medication use, COPD exacerbations and COPD symptoms over 12 weeks of treatment.

**Results:**

657 patients were randomized (glycopyrronium: 327; tiotropium: 330); 96% (630 patients) completed the study. Least squares mean trough FEV_1_ for both glycopyrronium and tiotropium was 1.405 L at Week 12, meeting the criterion for non-inferiority (mean treatment difference: 0 mL, 95% CI: –32, 31 mL). Glycopyrronium demonstrated rapid bronchodilation following first dose on Day 1, with significantly higher FEV_1_ at all time points from 0–4 h post-dose versus tiotropium (all p < 0.001). FEV_1_ area under the curve from 0–4 h (AUC_0–4h_) post-dose with glycopyrronium was significantly superior to tiotropium on Day 1 (p < 0.001) and was comparable to tiotropium at Week 12. Glycopyrronium demonstrated comparable improvements to tiotropium in TDI focal score, SGRQ total score, rescue medication use and the rate of COPD exacerbations (all p = not significant). Patients on glycopyrronium also had a significantly lower total COPD symptom score versus patients on tiotropium after 12 weeks (p = 0.035). Adverse events were reported by a similar percentage of patients receiving glycopyrronium (40.4%) and tiotropium (40.6%).

**Conclusion:**

In patients with moderate-to-severe COPD, 12-week blinded treatment with once-daily glycopyrronium 50 μg or tiotropium 18 μg, provided similar efficacy and safety, with glycopyrronium having a faster onset of action on Day 1 versus tiotropium.

**Trial registration:**

ClinicalTrial.gov, NCT01613326

## Background

Chronic obstructive pulmonary disease (COPD) is characterized by progressive airflow limitation that results in breathlessness, reduced exercise capacity, chronic cough and sputum production [1]. Inhaled bronchodilators, including long-acting muscarinic antagonists (LAMAs), have been shown to improve symptoms and health status, while reducing exacerbation rates, and are the cornerstone of pharmacological therapy for COPD [1].

Until recently, once-daily (o.d.) tiotropium was the only LAMA available for patients with COPD. Tiotropium is a well-known LAMA, is widely prescribed worldwide, and has been shown to improve lung function, dyspnea, exercise tolerance, and health status, while reducing acute exacerbations and potentially mortality, compared with placebo [1,2]. Two LAMAs, twice-daily (b.i.d.) aclidinium bromide and o.d. glycopyrronium (NVA237) have been recently approved for the management of COPD [3,4]. Both are presented in a dry-powder formulation [5,6].

In the Phase III GLycopyrronium bromide in COPD airWays 1, 2 and 3 (GLOW1, GLOW2 and GLOW3) studies in patients with moderate-to-severe COPD, glycopyrronium 50 μg o.d. demonstrated significantly improved bronchodilation, dyspnea, health status, rescue medication use and exercise tolerance, and reduced the risk of exacerbations, compared with placebo [7-9]. In the 52-week GLOW2 study, glycopyrronium was additionally evaluated against open-label (OL) tiotropium; the onset of bronchodilation with glycopyrronium was more rapid than that of OL tiotropium 18 μg o.d. and improvements in bronchodilation, dyspnea, health status, exacerbations and rescue medication use were comparable to those provided by OL tiotropium [9].

As the only once-daily LAMA available for comparison versus glycopyrronium, tiotropium is an appropriate control. However, due to technical difficulties, blinding tiotropium is challenging and therefore leads to studies utilizing OL designs [10]. Such studies, however, can introduce study bias in several respects. Patients will know they are on active treatment and therefore may potentially report treatment effects on symptoms and health outcomes more positively compared with placebo. In addition, study staff may introduce bias with regard to decisions affecting continuing study participation, concomitant medication use and adverse event responses [10]. The present GLOW5 study is the first study that compares glycopyrronium 50 μg o.d. with blinded tiotropium 18 μg o.d.; the objective of this study was to investigate the efficacy and safety of glycopyrronium versus blinded tiotropium in patients with moderate-to-severe COPD, over 12 weeks.

## Methods

### Patients

GLOW5 enrolled men and women ≥40 years of age, with moderate-to-severe stable COPD (Global Initiative for Chronic Obstructive Lung Disease [GOLD] Stage II or III according to the 2010 GOLD guidelines) [11], who were current or ex-smokers with a smoking history of at least 10 pack-years, and a post-bronchodilator forced expiratory volume in 1 second (FEV_1_) ≥30% and <80% of predicted and post-bronchodilator FEV_1_/forced vital capacity (FVC) <0.70 at screening. Post-bronchodilator refers to 45 minutes after inhalation of 84 μg ipratropium.

The main exclusion criteria were respiratory tract infection within 4 weeks prior to screening; COPD exacerbations requiring treatment with antibiotics and/or oral corticosteroids and/or hospitalization 6 weeks prior to screening; concomitant pulmonary diseases other than COPD; clinically significant cardiovascular disease (such as, but not limited to, unstable ischemic heart disease, New York Heart Association class III/IV left ventricular failure, myocardial infarction, arrhythmia [including paroxysmal atrial fibrillation]); history of asthma, diabetes, malignancy of any organ system, long QT syndrome or QTc >450 ms at screening, symptomatic prostatic hyperplasia, bladder-neck obstruction, moderate/severe renal impairment, urinary retention, narrow-angle glaucoma, a known history of alpha-1 antitrypsin deficiency; participation in the active phase of a supervised pulmonary rehabilitation program; and contraindications for tiotropium or ipratropium, or history of adverse reactions to inhaled anticholinergics.

All patients gave written, informed consent to participate in the study. The study protocol was reviewed and approved by Institutional Review Boards and ethics committees at participating centers (Additional file 1: Table S1).

### Study design and treatment

**Figure 1:**
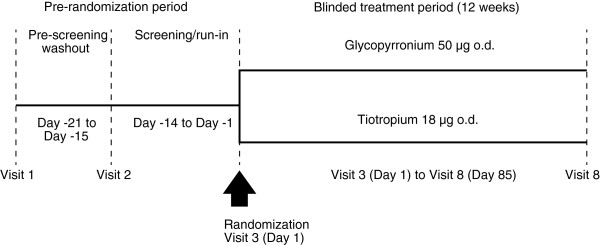
GLOW5 study design.

GLOW5 was a multicenter, blinded, double-dummy, parallel group, 12-week study. After a washout period (up to 7 days), followed by a 14-day run-in period, patients were randomized 1:1 to glycopyrronium 50 μg o.d. (delivered via the Breezhaler® device), tiotropium 18 μg o.d. (delivered via the HandiHaler® device), or matching placebos (Figure 1). Study drug was to be taken each morning between 08:00–11:00. Patients were to discontinue taking long-acting bronchodilator therapy before starting the run-in period (for at least 7 days for LAMAs and the long-acting β_2_-agonist [LABA] indacaterol, and for 48 h for other LABAs or LABA/inhaled corticosteroid [ICS] combinations). Patients on fixed-dose LABA/ICS combinations were switched to an equivalent dose of ICS contained in the fixed-dose combination. Patients were provided with a salbutamol/albuterol (short-acting β_2_-agonist; SABA) inhaler to be used as rescue medication during the study. They were instructed to abstain from taking rescue medication within 6 h of the start of each study visit.

### Treatment blinding

A double-dummy design was adopted in the study to achieve blinding. Following instruction on the correct use of the two devices, patients completed the inhalation of glycopyrronium or placebo to glycopyrronium via the Breezhaler® device followed as closely as they felt comfortable to do so by the inhalation of tiotropium or placebo to tiotropium via the HandiHaler® device, depending on their randomized treatment schedule. Study sites were instructed to ensure that glycopyrronium and placebo to glycopyrronium were only administered via the Breezhaler® device and that tiotropium and placebo to tiotropium were only administered via the HandiHaler® device. Additionally, blinding was achieved by specifying that the study medications be dispensed by a third party not involved in other aspects of the study, and by the use of study drugs that were similar in appearance, with the same schedule of administration.

An automated, interactive, voice-response technology was used to assign randomization numbers to patients who met the study criteria. Randomization numbers were used to link patients to treatment groups, and these were not communicated to the caller. Randomization data were kept strictly confidential until the time of unblinding, and were not accessible by anyone involved in the conduct of the study.

### Efficacy assessments

The primary efficacy objective of the study was to demonstrate the non-inferiority of glycopyrronium versus tiotropium for the parameter trough FEV_1_ (defined as the mean of the 23 h 15 min and the 23 h 45 min post-dose values), following 12 weeks of treatment. A key secondary objective was to demonstrate the superiority of glycopyrronium versus tiotropium on trough FEV_1_ after 12 weeks of treatment, if the primary objective of non-inferiority was demonstrated. Other secondary objectives were to evaluate the effect of glycopyrronium versus tiotropium on spirometric outcomes (FVC, peak FEV_1_, FEV_1_ area under the curve from 0–4 h [FEV_1_ AUC_0–4h_], inspiratory capacity [IC]), and on breathlessness measured using Transition Dyspnea Index (TDI) focal score, health status according to the St George’s Respiratory Questionnaire (SGRQ) total score, daily rescue medication use, COPD exacerbations and COPD symptoms over 12 weeks of treatment.

Pulmonary function assessments were performed using centralized spirometry. All spirometry assessments were reviewed centrally to ensure the maneuvers met the standards for repeatability and acceptability. The spirometer was customized and programmed according to the requirements of the study protocol in accordance with American Thoracic Society/European Respiratory Society standards [12], including predicted reference values. In order to reduce the variability of observations, the same equipment was used for all measurements during the study. Whenever possible, the same staff member coached and evaluated a patient at each visit. In addition, the spirometer was calibrated daily before measurements were made.

Spirometric measurements were taken prior to run-in to determine eligibility and to record post-bronchodilator FEV_1_ 45 minutes after inhalation of up to 84 μg ipratropium. Thereafter, spirometry was performed at randomization (Day 1) and Weeks 4 and 12. FEV_1_ and FVC were recorded at all clinic visits at the following time points relative to the morning dose: 45 and 15 min pre-dose, and 5, 15 and 30 min, 1 h, 23 h 15 min and 23 h 45 min post-dose on Day 1 and Weeks 4 and 12, and additionally at 2 h, 3 h and 4 h on Day 1 and Week 12. IC was also recorded at each clinic visit at 20 min pre-dose, 25 min, 2 h, 4 h and 24 h at Day 1 and Week 12.

Investigator-administered baseline dyspnea index (BDI) was assessed at Day 1, TDI at Weeks 4 and 12, and self-administered SGRQ was assessed at Day 1 and Week 12. All patients were provided with an electronic diary to record morning and evening symptoms twice daily. Patients recorded cough, wheezing, shortness of breath, sputum volume and color, night time awakenings and impact on daily activities, assigning a rating of 0 to 3 for each (0 being the best and 3 being the worst); the sum of these gave the total symptom scores (further details are included in Additional file 1: Table S2). Patients also recorded the use of rescue medication in their diary. Patient daily diaries were reviewed at Day 1 and Weeks 4 and 12 and features of COPD exacerbation and change in concomitant medication usage from baseline were noted.

COPD exacerbations were defined as worsening of two or more major symptoms (dyspnea, sputum volume or sputum purulence) for at least 2 consecutive days or worsening of any one major symptom together with any minor symptom (colds, fever without other cause, increased cough, increased wheeze or sore throat) for at least 2 consecutive days. Exacerbations were considered to be of moderate severity if they required treatment with systemic corticosteroids, antibiotics or both, and were considered severe if they also required hospitalization.

### Safety assessments

Safety was assessed by recording all treatment-emergent adverse events (AEs) and serious AEs (SAEs), monitoring vital signs and performing laboratory analyses (hematology, clinical chemistry and urinalysis). An AE was defined as the appearance or worsening of any undesirable sign, symptom, or medical condition occurring after starting the study drug, even if the event was not considered to be related to study drug. AEs were coded using the Medical Dictionary of Regulatory Activities (MedDRA) and summarized by primary system organ class, preferred term, maximum severity and relationship to study drug. An independent adjudication committee classified the reported serious cardio- and cerebro-vascular (CCV) events.

### Statistical analysis

Three populations were defined for the purpose of analysis. The full analysis set (FAS) included all randomized patients who received at least one dose of the study drug and was analyzed according to the allocated treatment group. The per-protocol set (PPS) included patients in the FAS who did not have major protocol deviations; patients were analyzed according to the treatment they were randomized to. Patients who did not take randomized treatment as per protocol in the 14 days prior to the trough assessment at Week 12 were excluded from the PPS. The safety set consisted of all patients who received at least one dose of study drug; patients were analyzed according to the treatment they received.

The primary analysis was performed in the PPS with imputation with last observation carried forward (LOCF), using a mixed model which contained treatment as a fixed effect, with the baseline measurement of FEV_1_ and FEV_1_ prior to and post inhalation of short-acting bronchodilator as covariates. The model also included smoking status at baseline (current/ex-smoker) and baseline ICS use (yes/no) and region as fixed effects and center nested within region as a random effect. The non-inferiority of glycopyrronium to tiotropium was claimed if the lower bound of the two-sided 95% CI for the treatment difference was greater than −50 mL.

If the primary objective of non-inferiority was met, then the superiority of glycopyrronium for trough FEV_1_ (imputed with LOCF) after 12 weeks of treatment was evaluated in the FAS using the same mixed model as specified for the primary analysis. Superiority could be demonstrated if the treatment difference in the FAS was statistically significant at the 5% level (two-sided) and the corresponding 95% CI lay entirely to the right (higher than) of 0 mL. Other secondary variables were analyzed in the PPS using the same mixed model as specified for the primary analysis, with the respective baseline values replacing baseline FEV_1_ as covariates. For each analysis, the estimated adjusted treatment difference for glycopyrronium minus tiotropium is displayed along with the associated 95% CI.

The analysis of rate of moderate or severe COPD exacerbations was based on a generalized linear model, assuming a negative binomial distribution. The model included treatment, smoking status at baseline, and baseline ICS use and region as fixed effects, with baseline total symptom score, COPD exacerbation history (the number of COPD exacerbations in the year before screening) and FEV_1_ prior to and post inhalation of short-acting bronchodilator as covariates. Log length of time in the study was included as an offset. All safety endpoints were summarized for the safety set.

### Sample size calculation

The non-inferiority margin for this study was specified as 50 mL. Based on the assumption that an improvement in FEV_1_ of approximately 100 mL is likely to be clinically relevant [13], a non-inferiority margin of 50% of this value i.e. 50 mL was considered appropriate. A total of 558 evaluable patients (279 per treatment group) would achieve a power of no less than 90% based on the following assumptions: a one-sided non-inferiority test comparing glycopyrronium to tiotropium with respect to mean trough FEV_1_ after 12 weeks of treatment at a significance level of 2.5%; a treatment difference of 0 mL in mean trough FEV_1_ after 12 weeks of treatment; a standard deviation of 200 mL; a non-inferiority margin (maximum allowable difference between the two treatment groups) of 50 mL in favor of tiotropium.

It was calculated that approximately 660 patients (330 per treatment group) would need to be randomized to make up for the loss of approximately 15% of patients due to major protocol deviations and drop-outs.

## Results

### Patient disposition and baseline characteristics

A total of 980 patients were screened, 657 patients were randomized (glycopyrronium: 327; tiotropium: 330; Figure 2); 96% (630 patients) completed the study. The percentage of patients who discontinued was similar in both groups. The two most common reasons for discontinuing treatment were withdrawal of consent and AEs.

**Figure 2:**
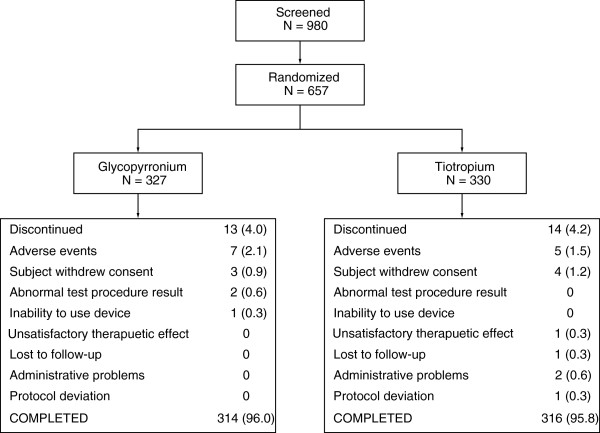
Patient disposition, n (%).

Baseline characteristics were similar between the treatment groups (Table 1). Mean age was 63.5 years, 73.8% of the patients were male, the majority were Caucasian (69.6%), and approximately one-half were ex-smokers. Most patients had moderate (58.4%) or severe (41.4%) COPD; one patient had mild COPD. The mean duration of COPD was 6.3 years. Approximately 23.6% of the patients had a documented history of exacerbations in the previous year. Mean post-bronchodilator FEV_1_ was 53.5% predicted and mean post-bronchodilator FEV_1_/FVC ratio was 47.3%.

**Table 1:** Baseline demographics and spirometry (safety set)

	**Glycopyrronium 50 μg o.d. (N = 327)**	**Tiotropium 18 μg o.d. (N = 330)**
Mean (SD) age, years	63.2 (7.9)	63.7 (8.0)
Male, n (%)	237 (72.5)	248 (75.2)
Ethnicity, n (%)		
Caucasian	225 (68.8)	232 (70.3)
Black	0	0
Asian	95 (29.1)	91 (27.6)
Native American	6 (1.8)	7 (2.1)
Other	1 (0.3)	0
Severity of COPD (GOLD 2010), n (%)		
Mild	0	1 (0.3)
Moderate	191 (58.4)	193 (58.5)
Severe	136 (41.6)	136 (41.2)
Mean (SD) duration of COPD, years	6.5 (5.1)	6.2 (5.1)
Baseline COPD exacerbation history*, n (%)		
0 exacerbations	255 (78.0)	247 (74.8)
1 exacerbation	52 (15.9)	61 (18.5)
≥2 exacerbations	20 (6.1)	22 (6.7)
ICS use at baseline, n (%)	163 (49.8)	174 (52.7)
Smoking history, n (%)		
Ex-smoker	179 (54.7)	182 (55.2)
Current smoker	148 (45.3)	148 (44.8)
Mean (SD) duration of smoking, pack-years	39.6 (20.4)	40.2 (21.5)
Mean (SD) FEV_1_ post-bronchodilator, L	1.5 (0.5)	1.5 (0.5)
Mean (SD) post-bronchodilator FEV_1_% predicted	53.2 (13.1)	53.9 (12.7)
Mean (SD) post-bronchodilator FEV_1_ reversibility, %	17.9 (13.5)	17.6 (13.6)
Mean (SD) post-bronchodilator FEV_1_/FVC, %	47.4 (10.7)	47.2 (10.5)

Pack-years = total years of smoking multiplied by cigarette packs smoked per day; *In the year prior to screening; FEV_1_ = forced expiratory volume in 1 second; FVC = forced vital capacity; SD = standard deviation; o.d. = once-daily.

### Spirometry

The least squares mean (LSM) trough FEV_1_ for both glycopyrronium and tiotropium was 1.405 L in the PPS after 12 weeks of treatment; the lower bound of the two-sided 95% CI for the treatment difference was higher than −50 mL, thus meeting the criterion for non-inferiority (LSM treatment difference: 0 mL, 95% CI: –32, 31 mL; one-sided p < 0.001; Table 2). Since the non-inferiority criterion was met, the superiority of glycopyrronium to tiotropium was tested for trough FEV_1_ after 12 weeks in the FAS, but no statistically significant difference was observed between the two treatment groups (mean difference 4 mL; p = 0.780; Table 2). The corresponding mean changes from baseline in trough FEV_1_ at Week 12 was 103 mL for glycopyrronium and 99 mL for tiotropium.

**Table 2:** Differences between treatment for primary and secondary efficacy outcomes (PPS)

**Variable**	**LSM (95% CI) treatment difference glycopyrronium versus tiotropium**	**p-value**
**Day 1**		
FEV_1_ 5 min post-dose, L	0.051 (0.036, 0.066)	<0.001
FEV_1_ 15 min post-dose, L	0.063 (0.046, 0.079)	<0.001
FVC 5 min post-dose, L	0.051 (0.013, 0.089)	0.008
FVC 15 min post-dose, L	0.050 (0.008, 0.092)	0.020
FVC 30 min post-dose, L	0.045 (0.001, 0.089)	0.046
Peak FEV_1_ (0–4 h), L	0.055 (0.034, 0.075)	<0.001
IC 30 min post-dose, L	0.078 (0.033, 0.123)	<0.001
IC 2 h post-dose, L	0.098 (0.045, 0.152)	<0.001
FEV_1_ AUC_0–4h_, L	0.058 (0.040, 0.076)	<0.001
**Week 12**		
Trough FEV_1_ (non inferiority; PPS; Primary objective)^†^, L	0 (−0.032, 0.031)	<0.001*
Trough FEV_1_ (superiority; FAS)^†^, L	0.004 (−0.025, 0.034)	0.780
FEV_1_ AUC_0–4h_, L	0.023 (−0.006, 0.053)	0.120
Peak FEV_1_ (0–4 h), L	0.025 (−0.005, 0.055)	0.107
IC 24 h post-dose, L	−0.034 (−0.101, 0.033)	0.318
TDI focal score	−0.188 (−0.614, 0.237)	0.385
SGRQ total score	0.65 (−1.19, 2.50)	0.488
**Over 12 weeks**		
Rescue medication use		
Change from baseline in mean daily number of puffs	0 (−0.3, 0.3)	0.852
Percentage of days with no rescue medication use	−1.5 (−6.2, 3.2)	0.528
Change from baseline in mean daily total symptom score^*¶*^	−0.3 (−0.5, 0.0)	0.035

Results of analysis in the per protocol set, unless otherwise stated; *One-sided p-value for the test of non-inferiority presented; ^†^Imputed with last observation carried forward; ^¶^Scored from 0–3 for both morning and evening symptoms (0 = lowest, 3 = highest; with the possible range of 0–18 for the daily score); AUC = area under the curve; CI = confidence interval; FAS = full analysis set; FEV_1_ = forced expiratory volume in 1 second; FVC = forced vital capacity; IC = inspiratory capacity; LSM = least squares mean; PPS = per-protocol set; TDI = Transition Dyspnea Index; SGRQ = St George’s Respiratory Questionnaire.

**Figure 3:**
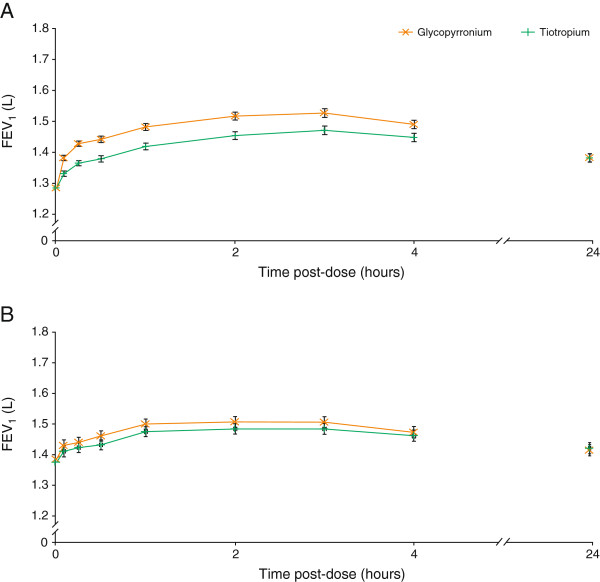
**FEV**_**1 **_**from 0 to 4 h and at 24 h on a) Day 1 and at b) Week 12 (PPS).** Data are LSM ± standard error; FEV_1_ = forced expiratory volume in 1 second; PPS = per-protocol set. **A**. p < 0.001 at all time points from 0–4 h; p = not significant at 24 h. **B**. p = not significant at all time points.

Following first dose on Day 1, significant differences in FEV_1_ were observed in favor of glycopyrronium, with LSM differences of 51 mL at 5 min and 63 mL at 15 min post-dose versus tiotropium (both p < 0.001; Table 2). Peak FEV_1_ and FEV_1_ AUC_0–4h_ post-dose in the glycopyrronium treatment group were significantly superior to the tiotropium group on Day 1 (both p < 0.001). FEV_1_ at all time points from 0–4 h were also significantly higher with glycopyrronium than with tiotropium on Day 1 (all p < 0.001; Figure 3A). FVC at Day 1 followed a similar pattern and was significantly higher with glycopyrronium than with tiotropium at post-dose time points of 5 min (LSM difference 51 mL; p = 0.008), 15 min (LSM difference 50 mL; p = 0.020) and 30 min (LSM difference 45 mL; p = 0.046; Table 2). On Day 1, IC was also significantly higher with glycopyrronium versus tiotropium at 30 min (p < 0.001) and 2 h (p < 0.001) post-dose.

**Figure 4:**
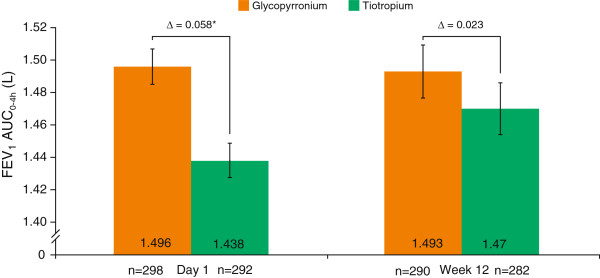
**FEV**_**1 **_**AUC**_**0–4h **_**treatment differences for glycopyrronium versus tiotropium on Day 1 and at Week 12 (PPS).** Data are LSM ± standard error; *p < 0.001; AUC = area under the curve; FEV_1_ = forced expiratory volume in 1 second; PPS = per-protocol set.

At Week 12, peak FEV_1_, FEV_1_ at all time points from 0–4 h and at 24 h (Figure 3B), and FEV_1_ AUC_0–4h_ (Figure 4) was comparable between glycopyrronium and tiotropium (all p = not significant [NS]). IC at 24 h post-dose at Week 12 was similar in the two treatment groups; change from baseline at all time points measured on Day 1 and Week 12 are presented in Table 3.

**Table 3:** Change from baseline and treatment differences (glycopyrronium vs tiotropium) in IC at all time points evaluated on Day 1 and Week 12 (PPS)

	**LSM (95% CI) treatment difference glycopyrronium versus tiotropium**	**Mean (SD) change from baseline (L)**	
**Time point**		**Glycopyrronium (N = 300)**	**Tiotropium (N = 293)**
**Day 1**			
25 min	0.078 (0.033,0.123)*	0.242 (0.252)	0.166 (0.236)
2 h	0.098 (0.045,0.152)*	0.298 (0.304)	0.197 (0.268)
4 h	0.035 (−0.021,0.090)	0.234 (0.284)	0.198 (0.307)
24 h	0.004 (−0.050,0.057)	0.117 (0.293)	0.111 (0.281)
**Week 12**			
−20 min	−0.029 (−0.097,0.038)	0.087 (0.369)	0.099 (0.355)
25 min	0.012 (−0.054,0.079)	0.211 (0.365)	0.187 (0.345)
2 h	0.055 (−0.014,0.124)	0.266 (0.368)	0.207 (0.385)
4 h	0.037 (−0.033,0.107)	0.233 (0.371)	0.186 (0.367)
24 h	−0.034 (−0.101,0.033)	0.126 (0.357)	0.148 (0.360)

IC = inspiratory capacity; SD = standard deviation; LSM = least squares mean; CI = confidence interval; *p < 0.001. There were no other statistically significant differences at any other time point measured.

### Symptoms, health status, exacerbations and diary card data

At Week 12, a comparable improvement was demonstrated by glycopyrronium and tiotropium in TDI focal score, with a non-significant LSM treatment difference (−0.188; p = 0.385; Table 2). TDI means (standard deviation) at Week 12 were 2 (2.90) points and 2.3 (3.0) points with glycopyrronium and tiotropium, respectively. A similar proportion of patients experienced a clinically meaningful improvement in TDI focal score (≥1 point) in both treatment groups (58.6%; odds ratio [OR] 1.06; p = 0.753) at Week 12 [14].

SGRQ total score at Week 12 was comparable between glycopyrronium and tiotropium, with a non-significant LSM treatment difference (0.65 points; p = 0.488; Table 2). Mean changes (improvements) from baseline were −5.60 and −7.25 with glycopyrronium and tiotropium, respectively. A comparable proportion of patients had a clinically meaningful improvement in the SGRQ total score (≥4 point reduction) at Week 12 in the glycopyrronium and tiotropium groups (55.2% and 54%, respectively; OR 1.11; p = 0.575) [15].

The number of patients who experienced a moderate or severe COPD exacerbation was small in both treatment groups; glycopyrronium (29 patients, 9.7%) and tiotropium (22 patients, 7.5%). Exacerbation rates also were low and similar in the subjects receiving glycopyrronium and tiotropium (0.38 exacerbations/year versus 0.35 exacerbations/year, respectively; rate ratio 1.10, 95% CI: 0.62, 1.93; p = 0.754). Other exacerbation-related endpoints are presented in the additional file 1: Figure S1.

The mean daily total COPD symptom score was statistically significantly lower with glycopyrronium compared with tiotropium, with a treatment difference of −0.3 (95% CI: –0.5, 0.0, p = 0.035; Table 2). Rescue medication use in the two treatment groups was comparable, with non-significant differences between the two treatment groups (Table 2).

### Safety

The overall incidence of AEs was similar between the two treatment groups (glycopyrronium 40.4%, tiotropium 40.6%; Table 4).The most frequently reported AE was COPD worsening, seen with a higher frequency in the tiotropium group (17.6%) compared with glycopyrronium (15.3%; Table 4). Of the other most frequently occurring AEs (at least three patients in either treatment group), only nasopharyngitis, headache, upper respiratory tract infection, and urinary tract infection occurred more frequently in the glycopyrronium group versus the tiotropium group (Table 4). AEs leading to discontinuation occurred in a comparable number of patients in both groups (Table 4).

**Table 4:** Most frequent AEs (at least three patients in either treatment group) and discontinuations due to AEs (safety set), n (%)

**Preferred term**	**Glycopyrronium 50 μg o.d. (N = 327) n (%)**	**Tiotropium 18 μg o.d. (N = 330) n (%)**
Any AE	132 (40.4)	134 (40.6)
COPD worsening	50 (15.3)	58 (17.6)
Nasopharyngitis	14 (4.3)	8 (2.4)
Headache	12 (3.7)	7 (2.1)
Bacterial upper respiratory tract infection	10 (3.1)	11 (3.3)
Upper respiratory tract infection	9 (2.8)	5 (1.5)
Cough	5 (1.5)	5 (1.5)
Viral upper respiratory tract infection	5 (1.5)	6 (1.8)
Urinary tract infection	4 (1.2)	1 (0.3)
Lower respiratory tract infection	3 (0.9)	3 (0.9)
Influenza	2 (0.6)	4 (1.2)
Non-cardiac chest pain	2 (0.6)	3 (0.9)
Pneumonia	2 (0.6)	3 (0.9)
Arthralgia	1 (0.3)	5 (1.5)
Dry mouth	1 ( 0.3)	5 (1.5)
Odema peripheral	1 ( 0.3)	5 (1.5)
Blood glucose increased	0	3 (0.9)
Gastritis	0	3 (0.9)
Renal failure acute	0	3 (0.9)
Sinusitis	0	3 (0.9)
Discontinuation due to AE(s)	7 (2.1)	5 (1.5)

AE = adverse event; o.d. = once daily.

SAEs occurred with a similar frequency in the glycopyrronium (3.4%) and tiotropium (3.9%) treatment groups. Infections and infestations were the most frequent SAEs; COPD worsening occurred more frequently in the tiotropium group (1.8%) than in the glycopyrronium group (0.9%). The proportion of patients with newly occurring or worsening clinically notable QTcF values was slightly higher with tiotropium (5.8%) compared with glycopyrronium (4.0%). Two patients in the glycopyrronium group had QTcF values >480 msec; none in the tiotropium group. The percentage of patients with an increase in QTcF from baseline of 30−60 msec were similar between the treatment groups (glycopyrronium 3.4%; tiotropium 3%). No patient had an increase from baseline in QTcF >60 msec.

The percentage of patients with cardio- and cerebro-vascular SAEs was similar between the two treatment groups (0.6%; Table 5). Two patients in the tiotropium group (0.6%; non-fatal stroke) and none in the glycopyrronium group had a major adverse cardiovascular event. There were no new onset atrial flutter events in either treatment group. One patient in the glycopyrronium group and none in the tiotropium group had a new-onset atrial fibrillation event. No deaths were reported in the study.

**Table 5:** CCV SAEs, n (%) (safety set)

	**Glycopyrronium 50 μg o.d. (N = 327) n (%)**	**Tiotropium 18 μg o.d. (N = 330) n (%)**
Patients with any serious CCV event	2 (0.6)	2 (0.6)
MACE	0	2 (0.6)
Non-fatal stroke	0	2 (0.6)
Non-major serious adverse cardiovascular events*	2 (0.6)	0

CCV = cardio- and cerebro-vascular; MACE = major cardiovascular adverse event; o.d. = once daily; SAE = serious adverse event; *Non-cardiac chest pain syndrome (n = 1), cardiac failure (n = 1).

## Discussion

Glycopyrronium, a once-daily LAMA in development, has undergone an extensive clinical development programme; results from three Phase III studies (GLOW1–3) have demonstrated that once-daily glycopyrronium 50 μg significantly improves lung function, dyspnea, health status, rescue medication use and exercise tolerance, and reduces the risk of exacerbations, versus placebo, with an acceptable safety profile [7-9].

Tiotropium is a key comparator in the evaluation of new bronchodilators; however, there are some challenges in using tiotropium as a control [10]. Since tiotropium cannot be easily blinded, several studies have used OL tiotropium as a control [16-19]. The GLOW2 study also evaluated glycopyrronium versus OL tiotropium [9]. Although GLOW2 was not powered to show statistical superiority of glycopyrronium over tiotropium, glycopyrronium was found to be comparable to tiotropium for all endpoints assessed. The impact of bias is a potential issue in an OL design, but it was minimized in the GLOW2 study by the use of objective spirometric endpoints. The purpose of the GLOW5 study was to allow a blinded comparison of the efficacy and safety of glycopyrronium to tiotropium in patients with moderate-to-severe COPD.

In the GLOW5 study, once-daily glycopyrronium demonstrated non-inferiority to once-daily blinded tiotropium in trough FEV_1_ at Week 12 in patients with moderate-to-severe COPD. In the daily symptom diaries, the total COPD symptom score was significantly lower in the glycopyrronium treatment group versus tiotropium (p = 0.035). Glycopyrronium and tiotropium demonstrated comparable exacerbation rates of 0.38 per year and 0.35 per year, respectively; this finding must be interpreted in the context of this being a 12-week study and that longer duration trials would normally be required for assessment of drug efficacy on exacerbation rates. Both glycopyrronium and tiotropium also similarly improved breathlessness and health status, and reduced rescue medication use; this is consistent with the results seen in the GLOW2 study [9]. Therefore, the closely similar effect of both treatments on lung function and clinical outcomes indicates that both treatments were comparable and similarly potent.

Similar to the results demonstrated in the GLOW2 study [9], in the current study, glycopyrronium provided rapid bronchodilation following first dose on Day 1, with significantly higher FEV_1_ at all time points from 0–4 h versus tiotropium (p < 0.001). A rapid onset of bronchodilation is a desirable feature in any COPD therapy. For patients with COPD, symptoms such as dyspnea and activity limitation are most challenging in the morning and reflect the greater morning burden of COPD [20,21]. The rapid onset of bronchodilation with glycopyrronium administered in the morning can be expected to have a positive impact on the morning routines and daily life of patients with COPD. Additionally, a faster onset of action is desirable, as long-term adherence to therapy may be lower for medications that do not have an immediate or direct effect on COPD symptoms [22]. Rapid onset of effect may lead to better long-term compliance to therapy which in turn has been shown to correlate with better treatment outcomes [23].

Both glycopyrronium and tiotropium had acceptable safety and tolerability profiles, with a comparable overall incidence of AEs between both treatment groups. Comparable safety of glycopyrronium and tiotropium was also observed in the GLOW2 study [9].

## Conclusion

The results from the 12-week GLOW5 study demonstrate that in patients with moderate-to-severe COPD, glycopyrronium 50 μg once daily provided similar efficacy and safety to tiotropium 18 μg once daily, with glycopyrronium providing a faster onset of action on Day 1 compared with tiotropium.

## Abbreviations

AEs: Adverse events; AUC: Area under the curve; BDI: Baseline dyspnea index; b.i.d: Twice-daily; CCV: Cardio- and cerebro-vascular; CI: Confidence interval; COPD: Chronic obstructive pulmonary disease; FAS: Full analysis set; FEV1: Forced expiratory volume in 1 second; FVC: Forced vital capacity; GLOW: GLycopyrronium bromide in COPD airWays; GOLD: Global Initiative for Chronic Obstructive Lung Disease; IC: Inspiratory capacity; ICS: Inhaled corticosteroid; LABA: Long-acting β_2_-agonist; LAMA: Long-acting muscarinic antagonist; LOCF: Last observation carried forward; LSM: Least squares mean; MedDRA: Medical Dictionary of Regulatory Activities; NS: Not significant; o.d.: Once-daily; OL: Open-label; OR: Odds ratio; PPS: Per-protocol set; SABA: Short-acting β_2_-agonist; SAE: Serious adverse events; SGRQ: St George’s Respiratory Questionnaire; TDI: Transition Dyspnea Index.

## Competing interests

KRC, in the last 3 years, has received compensation for consulting with Boehringer Ingelheim, CSL Behring, GlaxoSmithKline, Merck Frosst, Novartis, Takeda, Pfizer, Roche, Schering Plough and Grifols; has undertaken research funded by AstraZeneca, Boehringer Ingelheim, CSL Behring, Forest Labs, GlaxoSmithKline, Novartis, Parangenix, Roche, Takeda and Grifols; and has participated in continuing medical education activities sponsored in whole or in part by AstraZeneca, Boehringer Ingelheim, GlaxoSmithKline, Grifols, Merck Frosst, Novartis, Takeda and Pfizer. He is participating in research funded by the Canadian Institutes of Health Research operating grant entitled Canadian Cohort Obstructive Lung Disease (CanCOLD). He holds the GSK-CIHR Research Chair in Respiratory Health Care Delivery at the University Health Network, Toronto, Canada.

KMB, in the past 3 years, has received compensation for organizing or participating in advisory boards for Almirall Hermal, Cytos, Chiesi, Boehringer Ingelheim, AstraZeneca, Mundipharma, Novartis and Revotar Biopharmaceuticals and participated as a speaker in scientific meetings or courses supported by various pharmaceutical companies (Almirall Hermal, AstraZeneca, Boehringer Ingelheim, Novartis, Pfizer and Takeda), and has received consulting fees from Ablynx, Apellis Pharmaceuticals and Cytos. The institution where KMB is employed has received compensations for the design, performance or participation in single or multicentre clinical trials from several companies including Almirall, Boehringer Ingelheim, Cytos, GlaxoSmithKline, Mundipharma, Novartis, Pfizer, Revotar Biopharmaceuticals, Sterna AG, and TEVA.

JB has served as a consultant to Almirall, Novartis and Pfizer; been on advisory boards for Novartis, Almirall and Cytos; and received lecture fees from Novartis, Pfizer and Almirall, and her institution has received remuneration for participation in clinical trials sponsored by Novartis, Almirall, AstraZeneca, GlasxoSmithKline, Pfizer, Boehringer Ingelheim, Revotar, Cytos, Takeda, Merck, Teva, Mundipharma, Sterna and Infinity.

EDB has served as a consultant to AlkAbello, Almirall, Cephalon, Hoffman la Roche, ICON and MS Consulting Group; been on advisory boards for Almirall, AstraZeneca, Boehringer Ingelheim, Elevation Pharma, Forest, GlaxoSmithKline, Merck, Napp, Novartis and Nycomed; and received lecture fees from AlkAbello, AstraZeneca, Boehringer Ingelheim, Chiesi, GlaxoSmithKline, Novartis, Pfizer and Takeda; and his institution has received remuneration for participation in clinical trials sponsored by Actelion, Aeras, Almirall, AstraZeneca, Boehringer Ingelheim, Forest, GlaxoSmithKline, Hoffman La Roche, Merck, Novartis, Takeda and TEVA.

AD has received research, consulting, and lecturing fees from GlaxoSmithKline, Sepracor, Schering-Plough, Altana, Methapharm, AstraZeneca, ONO Pharmaceutical, Merck Canada, Forest Laboratories, Novartis, Boehringer Ingelheim Ltd, Pfizer Canada, SkyePharma, and KOS Pharmaceuticals.

RN, HC, MH, TO and PD are employees of Novartis and have no other conflicts of interest.

## Authors’ contributions

KRC, KMB, JB, EDB and AD contributed to the interpretation of data, revising of the manuscript at all stages and approved the final version. RN, HC, MH, TO and PD, as employees of the sponsor, contributed to the design and preparation, conduct, analysis and interpretation of the study. All authors read and approved the final manuscript. 

## Pre-publication history

The pre-publication history for this paper can be accessed here:

http://www.biomedcentral.com/1471-2466/14/4/prepub

## Supplementary Material

Additional file 1 Supplementary information. Click here for file
