# Supplementary material for: A blinded evaluation of the efficacy and safety of glycopyrronium, a once-daily long-acting muscarinic antagonist, versus tiotropium, in patients with COPD: the GLOW5 study
Source: BMC Pulm Med. 2014 Jan 17;14:4. doi: 10.1186/1471-2466-14-4 (PMC3907130; doi:10.1186/1471-2466-14-4)
Supplement: Additional file 1 — Supplementary information. [file 1471-2466-14-4-S1.docx]

**Additional File 1**

# A blinded evaluation of the efficacy and safety of glycopyrronium, a once-daily long-acting muscarinic antagonist, versus tiotropium, in patients with COPD: the GLOW5 study

Kenneth R. Chapman^1^; Kai-Michael Beeh^2^; Jutta Beier^2^; Eric D. Bateman^3^; Anthony D’Urzo^4^; Robert Nutbrown^5^; Michelle Henley^6^; Hungta Chen^6^; Tim Overend^5^; Peter D’Andrea^6^

*^1^Asthma and Airway Centre, University Health Network, Toronto; ^2^ Insaf Respiratory Research Institute, Wiesbaden, Germany; ^3^University of Cape Town, Cape Town, South Africa; ^4^Department of Family and Community Medicine, University of Toronto, Canada; ^5^Novartis Horsham Research Centre, West Sussex, UK; ^6^Novartis Pharmaceuticals Corporation, East Hanover, New Jersey, USA.*

**Assessments**

A secondary objective of the study was to evaluate the effect of glycopyrronium 50 µg once-daily (o.d.) versus tiotropium 18 µg o.d. on time to first moderate or severe chronic obstructive pulmonary disease (COPD) exacerbation.

**Statistical analysis**

Time to the first moderate or severe COPD exacerbation was displayed for each treatment

group with a Kaplan-Meier curve. The time to the first moderate or severe COPD exacerbation was analyzed using a Cox regression model stratified for region. The model included treatment, smoking status at baseline, and baseline inhaled corticosteroid (ICS) use as fixed effects and baseline total symptom score, COPD exacerbation history (the number of COPD exacerbations in the year before screening), forced expiratory volume in 1 second (FEV_1_) prior to and post inhalation of short acting bronchodilator as covariates. The estimated adjusted hazard ratio for glycopyrronium versus tiotropium is displayed along with the associated 95% confidence interval (CI) and two-sided p-value.

**Results**

There were no statistically significant differences between the two treatment groups with respect to time to first moderate or severe COPD exacerbation (hazard ratio 1.33, 95% CI: 0.76, 2.33; p=0.324; Supplemental Figure 1). This hazard ratio should be interpreted with caution since the proportional hazards assumption was not met. There were no differences between glycopyrronium and tiotropium in the proportion of patients with at least one moderate or severe COPD exacerbation leading to hospitalization (0.7% vs. 1.0%, respectively; odds ratio [OR] 0.79, 95% CI: 0.21, 2.94; p=0.728), treatment with systemic corticosteroids (6.0% vs. 6.2%, respectively; OR 1.06, 95% CI: 0.55, 2.04; p=0.873) or treatment with antibiotics (8.3% vs. 5.8%, respectively; OR 1.48, 95% CI: 0.77, 2.85; p=0.236).

**Supplemental Figure 1. Kaplan-Meier plot of the time to first moderate or severe exacerbation (per-protocol set; PPS).**

******

**Supplemental Table 1. List of Independent Ethics Committees or Institutional Review Boards by study center.**

| **Center Number** | **Ethics Committee or Institutional Review Board** | **Department/Organization** | **City, State/Province, Postal Code, Country** |
| --- | --- | --- | --- |
| 0020 | IRB Services |  | Aurora Ontario L4G0A5 Canada |
| 0021 | IRB Services |  | Aurora Ontario L4G0A5 Canada |
| 0023 | IRB Services |  | Aurora Ontario L4G0A5 Canada |
| 0024 | IRB Services |  | Aurora Ontario L4G0A5 Canada |
| 0025 | IRB Services |  | Aurora Ontario L4G0A5 Canada |
| 0026 | IRB Services |  | Aurora Ontario L4G0A5 Canada |
| 0031 | Agency for Medicinal Products and Medical Devices of Croatia | Central Ethics Committee | Zagreb Croatia |
| 0032 | Agency for Medicinal Products and Medical Devices of Croatia | Central Ethics Committee | Zagreb Croatia |
| 0033 | Agency for Medicinal Products and Medical Devices of Croatia | Central Ethics Committee | Zagreb Croatia |
| 0040 | Ethics Committee of the Institute Republic for Clinical and Experimental Medicine and Thomayer Hospital |  | Prague 4 140 59 Czech |
| 0041 | Ethics Committee of the Institute Republic for Clinical and Experimental Medicine and Thomayer Hospital |  | Prague 4 140 59 Czech |
| 0042 | Ethics Committee of the Institute Republic for Clinical and Experimental Medicine and Thomayer Hospital |  | Prague 4 140 59 Czech |
| 0043 | Ethics Committee of the Institute Republic for Clinical and Experimental Medicine and Thomayer Hospital |  | Prague 4 140 59 Czech |
| 0044 | Ethics Committee of the Institute Republic for Clinical and Experimental Medicine and Thomayer Hospital |  | Prague 4 140 59 Czech |
| 0045 | Ethics Committee of the Institute Republic for Clinical and Experimental Medicine and Thomayer Hospital |  | Prague 4 140 59 Czech |
| 0046 | Ethics Committee of the Institute Republic for Clinical and Experimental Medicine and Thomayer Hospital |  | Prague 4 140 59 Czech |
| 0047 | Ethics Committee of the Institute Republic for Clinical and Experimental Medicine and Thomayer Hospital |  | Prague 4 140 59 Czech |
| 0060 | Tallinn Medical Research Ethics Committee |  | Tallinn NA 11619 Estonia |
| 0061 | Tallinn Medical Research Ethics Committee |  | Tallinn NA 11619 Estonia |
| 0062 | Tallinn Medical Research Ethics Committee |  | Tallinn NA 11619 Estonia |
| 0063 | Tallinn Medical Research Ethics Committee |  | Tallinn NA 11619 Estonia |
| 0071 | CPP « Ile de France XI » Saint-Germain-Laye | Centre Hospitalier de Saint-Germain-Laye | Saint-Germain-Laye Cedex 78105 France |
| 0072 | CPP « Ile de France XI » - Saint-Germain-Laye | Centre Hospitalier de Saint-Germain-Laye | Saint-Germain-Laye Cedex 78105 France |
| 0073 | CPP « Ile de France XI » - Saint-Germain-Laye | Centre Hospitalier de Saint-Germain-Laye | Saint-Germain-Laye Cedex 78105 France |
| 0074 | CPP « Ile de France XI » Saint-Germain-Laye | Centre Hospitalier de Saint-Germain-Laye | Saint-Germain-Laye Cedex 78105 FRANCE |
| 0080 | Landesärztekammer Hessen Ethik-Kommission |  | Frankfurt am Main 60488, Germany |
| 0081 | Landesärztekammer Hessen Ethik-Kommission |  | Frankfurt am Main 60488, Germany |
| 0082 | Landesärztekammer Hessen Ethik-Kommission |  | Frankfurt am Main 60488, Germany |
| 0083 | Landesärztekammer Hessen Ethik-Kommission |  | Frankfurt am Main 60488, Germany |
| 0084 | Landesärztekammer Hessen Ethik-Kommission |  | Frankfurt am Main 60488, Germany |
| 0085 | Landesärztekammer Hessen Ethik-Kommission |  | Frankfurt am Main 60488, Germany |
| 0086 | Landesärztekammer Hessen Ethik-Kommission |  | Frankfurt am Main 60488, Germany |
| 0087 | Landesärztekammer Hessen Ethik-Kommission |  | Frankfurt am Main 60488, Germany |
| 0088 | Landesärztekammer Hessen Ethik-Kommission |  | Frankfurt am Main 60488, Germany |
| 0089 | Landesärztekammer Hessen Ethik-Kommission |  | Frankfurt am Main 60488, Germany |
| 0090 | Landesärztekammer Hessen Ethik-Kommission |  | Frankfurt am Main 60488, Germany |
| 0103 | Comité de Ética Independiente Zugueme |  | Guatemala City 01015 Guatemala |
| 0104 | Comité de Ética Independiente Zugueme |  | Guatemala City 01015 Guatemala |
| 0105 | Comité de Ética Independiente Zugueme |  | Guatemala City 01015 Guatemala |
| 0112 | Ethics Committee |  | Chennai Tamil Nadu, 600 087, India |
| 0113 | Institutional Human Research Ethics Committee | Karthick Polyclinic | Cimbatore, Tamil Nadu, 641 045, India |
| 0114 | KMCH Ethics Committee | Kovai Medical Center and Hospital | Coimbatore, Tamil Nadu 641 014, India |
| 0116 | Laitha Super Speciialities Hospital Ethics Committee |  | Guntur, Andhra Pradesh, 522 001, India |
| 0117 | Bangalore Central Ethics Committee |  | Bangalore, Karnataka, 560 084, India |
| 0118 | Sujalam Independent Ethics Committee | Associaton of Physicians of Ahmedabad | Ahmedabad, Gujarat, 380 009, India |
| 0119 | Midcity Independent Ethics Committee | Nagpur | Maharashtra, 440 009, India |
| 0150 | IRB of the Catholic Univ. og Korea St. Paul's Hospital |  | Dongdaemun-gu Seoul S Korea |
| 0151 | IRB of Yeungnam University Medical Center |  | Nam-gu Daegu 705-717 Korea |
| 0152 | IRB of Kyunghee Univ. Hospital |  | Dongdaemun-gu Seoul 130-702 Korea |
| 0153 | IRB of Pusan National University Hospital |  | Pusan 602-739 Korea |
| 0154 | IRB of Korea University Guro Hospital |  | Guro-gu Seoul 152-703 Korea |
| 0155 | IRB of Soonchunhyang University Bucheon Hospital |  | Wonmi-gu, Bucheon-si Gyeonggi-do 420-767 S Korea |
| 0156 | IRB of Chungang University Hospital |  | Dongjak-gu Seoul S Korea |
| 0181 | Ethics Committee for Clinical Research at P. Stradins Clinical University Hospital Development Society |  | Riga LV-1002, Latvia |
| 0182 | Ethics Committee for Clinical Research at P. Stradins Clinical University Hospital Development Society |  | Riga LV-1002, Latvia |
| 0191 | Lithuanian Bioethics Committee |  | Vilnius, LT-01128, Lithuania |
| 0192 | Lithuanian Bioethics Committee |  | Vilnius, LT-01128, Lithuania |
| 0193 | Lithuanian Bioethics Committee |  | Vilnius, LT-01128, Lithuania |
| 0194 | Lithuanian Bioethics Committee |  | Vilnius, LT-01128, Lithuania |
| 0195 | Lithuanian Bioethics Committee |  | Vilnius, LT-01128, Lithuania |
| 0196 | Lithuanian Bioethics Committee |  | Vilnius, LT-01128, Lithuania |
| 0197 | Lithuanian Bioethics Committee |  | Vilnius, LT-01128, Lithuania |
| 0200 | Manila Doctors Hospital Institutional Review Board |  | Ermita, Manila, Philippines |
| 0201 | Veterans Memorial Medical Center Institutional Review Board |  | Diliman, Quezon City, Philippines |
| 0202 | University of the Philippines Manila Research Ethics Board |  | Ermita, Manila, Philippines |
| 0203 | Mary Mediatrix Medical Center Institutional Review Board |  | Lipa City, Batangas, Philippines |
| 0210 | Komisja Bioetyczna Śląska Izba Lekarska |  | Katowice 40-126 Poland |
| 0211 | Komisja Bioetyczna przy Dolnośląskiej Izbie Lekarskiej |  | Wrocław 50-333 Poland |
| 0212 | Komisja Bioetyczna przy OIL Gdańsk |  | Gdańsk 80-204 Poland |
| 0213 | Komisja Bioetyczna przy Donośląskiej Izbie Lekarskiej |  | Wrocław 50-333 Poland |
| 0214 | Komisja Bioetyczna przy Donośląskiej Izbie Lekarskiej |  | Wrocław 50-333 Poland |
| 0230 | Human Research Ethics Committee, Faculty of Health Sciences | Groote Schuur Hospital | Observatory 7925 South Africa |
| 0231 | SAMA Research Ethics Committee, Castle Walk Office Park | Erasmuskloof | Pretoria 0153 South Africa |
| 0236 | SAMA Research Ethics Committee, Castle Walk Office Park | Erasmuskloof | Pretoria South Africa |
| 0251 | Chang Gung Memorial Hospital Institutional Review Board |  | Taipei City 10507 Taiwan |
| 0252 | Institutional Review Board China Medical University Hospital |  | Taichung city NA 40447 Taiwan |
| 0253 | Chang Gung Memorial Hospital Institutional Review Board |  | Taipei City NA 10507 Taiwan |
| 0254 | Far Eastern Memorial Hospital Institutional Review Board |  | New Taipei City NA 22060 Taiwan |

**Supplemental Table 2. Information captured in the patient diary.**
